# Syncope Linked to QT‐Interval Prolongation and Global T‐Wave Inversion: A Clinical Case of Acute Pulmonary Embolism

**DOI:** 10.1111/anec.70049

**Published:** 2025-02-14

**Authors:** Xue‐Yan Zhang, Jing‐Xiu Li, Min Gao, Xue‐Qi Li, Ming‐Yu Zhang

**Affiliations:** ^1^ Department of Cardiology The Fourth Affiliated Hospital of Harbin Medical University Harbin China; ^2^ Department of Electrocardiography, Division of Life Sciences and Medicine, The First Affiliated Hospital of USTC University of Science and Technology of China Hefei Anhui China

**Keywords:** case report, pulmonary embolism, QT‐interval prolongation, T‐wave inversion

## Abstract

The incidence and mortality rates of acute pulmonary embolism (APE) are high in clinical emergencies, making early diagnosis and risk stratification crucial. Electrocardiogram (ECG) plays a significant role in guiding the diagnosis and differential diagnosis of pulmonary embolism. Acute pulmonary embolism can present with various ECG manifestations. The presence of pulmonary hypertension and increased right ventricular load in pulmonary embolism can lead to T wave inversion in the right cardiac lead. Additionally, some patients may exhibit a prolonged QT interval, which is associated with the pathophysiological processes resulting from both pulmonary hypertension and myocardial ischemia.

## Graphical abstract

Background: Acute pulmonary embolism (APE) is characterized by high incidence, high mortality, and high misdiagnosis rate. Most symptomatic patients with APE have corresponding changes in electrocardiogram, which should be carefully observed and combined with clinical conditions is helpful for diagnosis. Case summary: A 70‐year‐old patient with chest pain and syncope for 1 day presented to the emergency department. The electrocardiogram suggested prolonged QT interval and inversion of prethoracic lead T‐wave. After improving the relevant imaging examination, it was acute pulmonary embolism, and anticoagulant treatment was given for several days, and the QT interval returned to normal. Discussion: Sometimes, it is hard to distinguish acute pulmonary embolism from acute coronary syndrome and aortic dissection because of similar manifestations. Various imaging tests can confirm the diagnosis. These diseases often lead to the appearance of T‐wave inversion and prolonged QT interval in the electrocardiogram. However, a careful analysis of the electrocardiogram can further clarify the corresponding diagnosis.

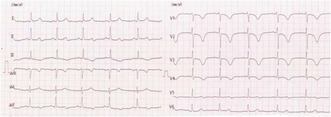

## 1 Case Presentation

**FIGURE 1:**
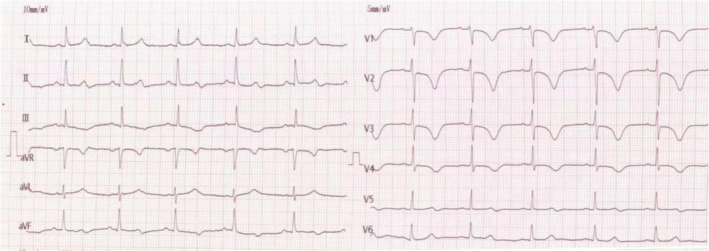
Findings of ECG performed at admission. Initial ECG revealed sinus rhythm, with a hear rate of 68 beats/min. QT‐interval prolongation to 520 ms, hear rate‐corrected QT‐interval prolongation to 540 ms, and T‐wave inversion in lead II III AVF, and V1–V4.

A 70‐year‐old patient with chest pain and syncope for 1 day presented to the emergency department of the Fourth Affiliated Hospital of Harbin Medical University. He had a history of hypertension for 20 years. There was no family history of sudden cardiac death. Physical examination revealed a heart rate of 65 beats/min and a blood pressure of 167/100 mmHg. An electrocardiogram (ECG) on admission is shown in Figure 1. Laboratory values were troponin I 0.068 ng/mL (0–0.034 ng/mL), creatine kinase‐MB 1.42 ng/mL (0–3.38 ng/mL), N‐terminal pro‐B‐type natriuretic peptide 5140 pg/mL (0–326 pg/mL), and D‐dimer 3.04 mg/L (0–0.55 mg/L). An arterial blood gas showed pH 7.44, pCO_2_ 36 mmHg, pO_2_ 74 mmHg.

Question: What does the ECG demonstrate? How should this patient be treated?

## 2 Clinical Course

**FIGURE 2:**
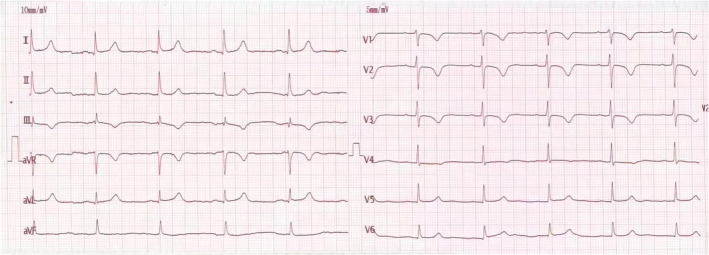
Findings of ECG performed at discharge ECG showed the QT interval had shortened to 400 ms.

The provisional diagnosis was acute coronary syndrome. However, findings of coronary computed tomography angiography showed left main coronary artery mild stenosis and left anterior descending artery mild stenosis. Echocardiography revealed systolic pulmonary artery pressure 55 mmHg. Venous Doppler ultrasonography was positive for deep venous thrombosis. The patient underwent computed tomography pulmonary angiography which revealed pulmonary embolism (PE). He was anticoagulated with low‐molecular‐weight heparin for 6 days. Repeated ECG showed that QT interval had shortened to 400 ms (Figure 2). The patient recovered favorably and was discharged from hospital.

## 3 Discussion

Syncope is observed in approximately 10% of patients as an initial presentation of pulmonary embolism (PE) (Castelli et al. 2003). It is recognized as a significant risk factor for sudden cardiac death (SCD) in various clinical contexts. Careful differentiation of electrocardiographic (ECG) findings in individuals presenting with syncope is crucial to exclude structural heart diseases, such as cardiomyopathy, and conduction system abnormalities, including atrioventricular block. In this case, no structural abnormalities were detected on echocardiography, and the electrocardiogram did not reveal arrhythmias. Furthermore, the left ventricular ejection fraction was within normal limits, rendering cardiomyopathy and arrhythmia unlikely causes of syncope. In the context of PE, syncope is primarily attributed to mechanisms such as a vasovagal response or acute right ventricular pressure overload, which may lead to right ventricular failure.

Acute episodes of syncope accompanied by QT prolongation on an electrocardiogram (ECG) are often suggestive of long QT syndrome (LQTS). Without comprehensive evaluation, there is a risk of misdiagnosing individuals with LQTS. LQTS is a genetic primary arrhythmia syndrome associated with potentially life‐threatening arrhythmias, such as torsades de pointes, and an increased risk of sudden cardiac death. However, QT prolongation can also result from various secondary causes, including electrolyte imbalances, pulmonary embolism, and the use of specific medications such as class I and III antiarrhythmics, macrolide antibiotics, arsenic trioxide, antimalarial agents, antipsychotics, and methadone. In this case, an evaluation of the patient's family and medical history, along with normal serum electrolyte levels, effectively ruled out these secondary causes.

Precordial T‐wave inversion is commonly indicative of right ventricular hypertrophy and overload. A precordial T‐wave inversion on ECG may result from various diseases, including myocardial ischemia (e.g., Wellens criteria), stress cardiomyopathy, post‐pacing, hypertrophic cardiomyopathy, subarachnoid hemorrhage, and pulmonary embolism. Wellens' syndrome is defined by inverted T‐waves in leads I, aVL, and V2–V5 on an ECG, signifying significant stenosis of the proximal left anterior descending coronary artery. Additionally, coronary computed tomography angiography indicates modest stenosis in the left major coronary artery and the left anterior descending artery. These characteristics can effectively separate pulmonary embolism from Wellens' syndrome. T wave inversion and QT prolongation may be linked to myocardial ischemia induced by catecholamine or histamine activity (Lui 1993; Zhao, Wang, and Wang 2015). Following the physical examination and supplementary investigation results, we ruled out subarachnoid hemorrhage and post‐pacing T‐wave memory as etiologies for the inverted T‐waves.

Physicians must remain alert for symptoms like syncope, T‐wave inversions, and simultaneous QT‐interval prolongation in the inferior and anterior leads as possible signs of pulmonary embolism.

## Author Contributions

X.‐Y.Z., J.‐X.L., M.G., and X.‐Q.L. contributed significantly to data collection and manuscript preparation. X.‐Y.Z. and M.‐Y.Z. performed the analysis with discussion.

## Conflicts of Interest

The authors declare no conflicts of interest.

## Data Availability

The data that support the findings of this study are openly available in Zhao, Wang, and Wang (2015).

## References

[anec70049-bib-0001] Castelli, R. , P. Tarsia , C. Tantardini , G. Pantaleo , A. Guariglia , and F. Porro . 2003. “Syncope in Patients With Pulmonary Embolism: Comparison Between Patients With Syncope as the Presenting Symptom of Pulmonary Embolism and Patients With Pulmonary Embolism Without Syncope.” Vascular Medicine 8, no. 4: 257–261. 10.1191/1358863x03vm510oa. 15125486

[anec70049-bib-0002] Lui, C. Y. 1993. “Acute Pulmonary Embolism as the Cause of Global T Wave Inversion and QT Prolongation. A Case Report.” Journal of Electrocardiology 26, no. 1: 91–95. 10.1016/0022-0736(93)90070-t. 8433060

[anec70049-bib-0003] Zhao, Y. T. , L. Wang , and B. Wang . 2015. “Syncope With QT Interval Prolongation and T‐Wave Inversion: Pulmonary Embolism.” American Journal of Emergency Medicine 33, no. 10: 1546.e5–6. 10.1016/j.ajem.2015.07.023. 26286817

